# Parent and Caregiver Willingness for Childhood COVID-19 Vaccination in Urban Uganda: Applying the Behavioral and Social Drivers (BeSD) Framework

**DOI:** 10.7759/cureus.111886

**Published:** 2026-07-01

**Authors:** Evanka Annyapu, Ronald Anguzu, Madeline Gwinn, Harriet Babikako, Jenna Loewus, Gouri Bollepalli, Anvika Annyapu, Kamulegeya John, Arthur Kiconco, Laura Cassidy

**Affiliations:** 1 Institute for Health and Humanity, Medical College of Wisconsin, Milwaukee, USA; 2 Department of General Medicine, Child and Family Foundation Uganda, Kawempe, UGA; 3 Department of Epidemiology and Biostatistics, Makerere University School of Public Health, Kampala, UGA; 4 Child Health and Development Center, Makerere University College of Health Sciences, Kampala, UGA; 5 College of Letters and Sciences, University of Wisconsin, Madison, USA; 6 Department of Epidemiology and Public Health, African Field Epidemiology Network (AFENET), Kampala, UGA

**Keywords:** behavioral and social drivers framework, childhood vaccinations, covid-19 vaccinations, uganda, vaccine coverage

## Abstract

Introduction: Vaccinations are widely recognized for protecting children from infectious diseases. During the COVID-19 pandemic, immunization efforts became especially important. Despite these efforts, achieving high vaccine uptake has remained challenging in Uganda, underscoring the need to better understand the factors that influence caregivers’ decisions regarding childhood COVID‑19 vaccination. The acceptability of routine childhood COVID-19 vaccine boosters among parents or caregivers in Uganda remains uncertain. This study examined factors associated with parent/caregiver willingness to vaccinate their children against COVID-19 in an urban immunization clinic in Kampala, Uganda.

Methods: We conducted a cross-sectional study among 230 caregivers with children under 59 months of age in an urban postnatal clinic in Kawempe Division, Kampala, Uganda, from June 2023 to July 2023. The outcome was willingness to receive annual routine COVID-19 vaccination for their child. Covariate selection was informed by the World Health Organization (WHO) Behavioral and Social Drivers (BeSD) framework: (i) thoughts and feelings (disease risk perception, confidence in vaccine safety, and benefits), (ii) social processes (family and friend norms), and (iii) practical issues (affordability). Modified Poisson regression identified factors independently associated with willingness to receive routine COVID-19 vaccination for their children. Adjusted prevalence rate ratios (adj. PRR) and 95% confidence intervals (95% CI) were calculated. P-values of <0.05 were considered statistically significant.

Among 230 respondents, 83.9% (n = 193) reported willingness to vaccinate their children against COVID-19, with female caregivers aged 15-24 years comprising 51% (n = 117) of our study sample. After controlling for confounders, willingness was higher among those with moderate confidence in vaccine benefits (PRR: 3.07; 95% CI: 1.37-6.85; p = 0.006) and very important confidence in vaccine benefits (PRR: 3.11; 95% CI: 1.41-6.89; p = 0.005), positive family norms (PRR: 1.35; 95% CI: 1.04-1.75; p = 0.022), and intention to vaccinate themselves (PRR: 2.68; 95% CI: 1.18-6.07; p = 0.019).

Conclusion: Targeting BeSD factors related to confidence in vaccine benefits, family norms, and parent/caregivers’ vaccination intentions could help address barriers to routine childhood COVID-19 vaccination, potentially improving COVID-19 vaccination rates among urban, healthcare-seeking caregivers in similar study settings.

## Introduction

Vaccination has been proven to be one of the most cost-effective public health interventions, capable of preventing up to three million deaths annually and significantly lowering childhood mortality rates [1]. Since the World Health Organization (WHO) declared COVID-19 a global pandemic on March 11, 2020 [2], over 777 million cases and 7.1 million deaths have been reported worldwide as of January 12, 2025 [3]. Children infected with COVID-19 accounted for 1% of global cases early in the pandemic [4]. However, with the Delta and Omicron variants, infections in children became more frequent and severe [5]. By 2022, during an Omicron surge, children made up 12.9% of global cases [3]. Immunizations help prevent a quarter of the eight million deaths and related disabilities among children under five years of age, underscoring their immense importance. Since the rollout of COVID-19 vaccines on July 22, 2020, 67% of the global population has completed the primary vaccination series, with 32% receiving at least one booster dose [6]. In Uganda, 44% of the population has received at least one COVID-19 vaccine dose [6].

Implementation gaps in immunization uptake persist for COVID-19 and routine immunization, especially in Sub-Saharan Africa (SSA), including Uganda, where millions of children fail to complete vaccination schedules. In Uganda, the third doses of vaccines such as diphtheria-tetanus-pertussis (DTP3), polio, and pneumococcal conjugate vaccine (PCV) are underutilized [7]. Low vaccination rates contribute to an alarming 24,000 preventable child deaths daily around the world, with 17% of the deaths among children under five linked to vaccine-preventable diseases [1].

The COVID-19 pandemic exacerbated this challenge by disrupting routine healthcare delivery, including immunization services. Vaccine hesitancy has been a common obstacle worldwide. There are low vaccine acceptance rates in the Middle East, Russia, and parts of Africa. However, there remains a need for more studies in SSA to understand the breadth of vaccine hesitancy [8]. With the introduction of COVID-19 vaccinations, Uganda experienced significant challenges in maintaining its regular vaccination schedules. Although COVID-19 vaccines have been rolled out in Uganda, coverage remains low compared to other countries in SSA, and even lower compared to high-income countries [9]. Furthermore, the willingness of Ugandans to integrate annual COVID-19 booster doses into regular childhood vaccination schedules remains uncertain.

Several factors contribute to low childhood COVID-19 vaccination rates in Uganda, including misinformation about vaccine safety and efficacy [10]. Caregivers’ skepticism about side effects, vaccine administration, and mistrust of healthcare systems exacerbate the problem [11]. Sociocultural dynamics, including limited male partner involvement and discouragement by some community leaders, reduce caregiver participation in child immunizations. Policy responses to these challenges include interventions by global organizations like the WHO and Uganda’s Ministry of Health (MoH). Programs such as the Expanded Program on Immunization (EPI) aim to improve vaccine coverage. As of January 12, 2025, the proportion of persons vaccinated with at least one COVID-19 dose was 44% [6].

There is a paucity of published evidence regarding the specific factors influencing caregivers’ acceptance of routine COVID-19 vaccinations for their children in Uganda [12-13]. While vaccine hesitancy is well-documented, understanding the contextual factors underlying these attitudes and behaviors informed by novel frameworks such as the behavioral and social drivers (BeSD) framework, among others, is limited. This study explored the factors influencing caregivers’ willingness to accept COVID-19 vaccinations for their children in Kawempe Division in Kampala, Central Uganda. Confidence in the benefits of the vaccine, family norms, and caregivers' own intentions to receive vaccinations were evaluated to inform future targeted interventions to increase vaccine uptake. This article was previously presented as a meeting abstract at the 2024 Consortium of Universities Global Health Meeting on March 8, 2024. 

## Materials and methods

Study design and setting

This cross-sectional study was conducted in Kawempe Division, which is one of five administrative divisions of Kampala District, the capital city of Uganda. Kampala District has an estimated total population of 1,793,600 with 52.6% females, while Kawempe Division, an urban setting, has an estimated total population of 403,000 with 53.1% females [14]. This health facility-based study was conducted in the postnatal/immunization clinic of the Child and Family Foundation (CFU) Medical Center, a private-not-for-profit health center in an urban community of Kawempe Division.

Sample size, sampling strategy, and data collection

A purposive sample of caregivers with children less than 59 months of age attending the CFU clinic to receive postnatal and/or immunization services was eligible for inclusion. Participants were consecutively selected at the postnatal/immunization clinic at the CFU Medical Center in Kawempe Division, Kampala, between June and July 2023. Selected participants were approached for participation after completing general group health education sessions at the CFU outpatient clinic. Data for the current study on caregiver willingness to vaccinate children against COVID-19 and routine immunization outcomes were collected as secondary outcomes for the primary study among postpartum adolescents described in detail elsewhere [15]. Prior sample size calculations for the primary study estimated that 227 participants were required to detect associations between intimate partner violence (40%) and major depressive disorder (MDD), assuming a 6% MDD prevalence, 40% IPV exposure, 80% power, and an alpha of 0.05. The consecutively sampled caregivers, namely, 230 participants, were adequately powered to examine the immunization outcomes reported in the current study. Trained research assistants administered interviewer-administered questionnaires to collect sociodemographic data and BeSD framework variables. Data were entered into REDCap, a secure HIPAA-compliant platform, and exported to STATA version 18.0 (Released 2023; Statistical Software, StataCorp LLC; College Station, TX, USA) for analysis.

Study measures

Our outcome categorical variable was willingness to vaccinate their children against COVID-19 (yes/no). The outcome was assessed using the item: "Would you want your child to receive the COVID-19 vaccine?" with response options: "No, you do not want to," "Yes, you do want to," and "Not sure" (Appendix Supplementary Table 1). This was dichotomized into "Yes" versus "No/Not sure for the regression analysis. This item referred to COVID-19 booster vaccines, which were reported to reduce the severity of COVID-19 disease in adults, and not in children less than five years of age.

The key independent variables were adopted from the WHO Behavioral and Social Drivers (BeSD) framework domains, which included thoughts and feelings (disease risk perception, confidence in vaccine safety, and benefits), social processes (family and friend norms), and practical issues (availability, affordability, and ease of access). The BeSD variables included confidence in vaccine benefits (thinking and feelings about disease risk perception, confidence in vaccine safety, and benefits), family and friend norms (social processes), and vaccine affordability in terms of cost of receiving the COVID-19 vaccine (practical issues). The outcome and key independent items are publicly available from the WHO guidebook that supports the use of the BeSD of vaccination tools to understand what drives uptake of vaccines [16].

Categorical covariates included sociodemographic characteristics, namely, caregivers’ age category in years (15-24, 25-34, and 35-49 years), caregivers’ gender (male/female), employed (yes/no), marital status (single, married, previously), education level (none, primary, secondary or more), household wealth index/quintile (poorest, poor, medium, wealthy, wealthiest), and caregivers’ willingness to receive COVID-19 vaccine (yes/no). Continuous variables included gravidity (number of children ever born regardless of birth outcome).

Data analysis

Study data were entered into REDCap^TM^, a secure, HIPAA-compliant system. De-identified data were exported to STATA version 18.0 for statistical analyses. Descriptive analyses were conducted for summary statistics reporting frequencies and percentages for categorical variables and means or medians for continuous variables with corresponding standard deviations (SD) or interquartile ranges (IQR), respectively. Modified Poisson regression analyses tested the independent association between behavioral and social factors and willingness to receive childhood COVID-19 vaccination while controlling for potential confounding factors. Statistical significance was considered at p-values of <0.05. Prevalence rate ratios (PRR) and 95% confidence intervals (95% CI) were reported as appropriate measures of association since our outcome variable was common, i.e., prevalence >10%. Adjusted PRRs were computed for independent associations between willingness to receive childhood COVID-19 vaccination and covariates using generalized linear model (GLM) analysis with robust standard errors. Independent variables with a p ≤ 0.10 and biologically plausible factors were included in the multivariable modified Poisson regression models to identify factors independently associated with willingness to receive annual childhood COVID-19 vaccination. Assumptions for Poisson regression analyses were met. This research was approved by both the Medical College of Wisconsin (MCW) and the AIDS Support Organization (TASO) Institutional Review Boards. 

## Results

A total of 230 child caregivers participated in the study, and 83.9% (n = 193) were willing to vaccinate their children against COVID-19. Caregivers’ mean ± SD age was 25.6 ± 6.1 years, 50.9% (n = 117) of caregivers were aged 15-24 years, 30.9% (n = 71) had the poorest household wealth index, and 74% (n = 170) were unemployed (Table 1).

**Table 1:** Descriptive statistics for routine childhood vaccination * Fisher's exact test; ** Chi-square test for categorical variables;*** t-tests for continuous variables. Significance: p < 0.05

		Outcome: Intention for child to receive COVID-19 vaccination			
Characteristic	Total, N (%)	No, n (%)	Yes, n (%)	Test	p-value
		37 (16.1)	193 (83.9)		
Caregiver’s age, years*					
35-49	25.0 (10.9)	2.0 (5.4)	23.0 (11.9)	1.514	0.537
25-34	88.0 (38.3)	14.0 (37.8)	74.0 (38.3)		
15-24	117.0 (50.9)	21.0 (56.8)	96.0 (49.7)		
Sex of child**					
Male	101.0 (43.9)	15.0 (40.5)	86.0 (44.6)	0.253	0.726
Female	127.0 (55.2)	22.0 (59.5)	105.0 (54.4)		
Missing	2.0 (0.9)	0.0 (0.0)	2.0 (1.0)		
Employment**					
Yes	60.0 (26.1)	10.0 (27.0)	50.0 (25.9)	0.020	0.887
No	170.0 (73.9)	27.0 (73.0)	143.0 (74.1)		
Marital status**					
Single	35.0 (15.2)	6.0 (16.2)	29.0 (15.0)	0.062	0.969
Married	147.0 (63.9)	23.0 (62.2)	124.0 (64.3)		
Previously married	48.0 (20.9)	8.0 (21.6)	40.0 (20.7)		
Education*					
None	16.0 (7.0)	0.0 (0.0)	16.0 (8.3)	3.684	0.152
Primary	108.0 (47.0)	17.0 (46.0)	91.0 (47.2)		
Secondary	106.0 (46.1)	20.0 (54.1)	86.0 (44.6)		
Household wealth quintile**					
Poorest	71.0 (30.9)	7.0 (18.9)	64.0 (33.2)	3.339	0.503
Poor	41.0 (17.8)	7.0 (18.9)	34.0 (17.6)		
Medium	62.0 (27.0)	12.0 (32.4)	50.0 (25.9)		
Wealthy	34.0 (14.8)	6.0 (16.2)	28.0 (14.5)		
Wealthiest	22.0 (9.6)	5.0 (13.5)	17.0 (8.8)		
Caregiver's intent to receive COVID-19 vaccine*					
No	19.0 (8.3)	15.0 (40.5)	4.0 (2.1)	60.625	<0.001
Yes	211.0 (91.7)	22.0 (59.5)	189.0 (97.9)		
Caregiver's age, years***					
Mean ± SD	25.6 ± 6.1	24.7 ± 6.2	25.8 ± 6.0	-1.033	0.303
Child's age, months***					
Mean ± SD	19.2 ± 13.8	17.7 ± 12.2	19.5 ± 14.1	-0.736	0.462
Gravidity***					
Mean ± SD	2.4 ± 1.6	2.1 ± 1.3	2.5 ± 1.7	-1.223	0.223

Among the 230 child caregivers, 93.1% (n = 214) of their children had received all routine childhood vaccinations. There were no statistically significant differences between socioeconomic factors, including maternal age, education, or household wealth and receipt of routine vaccination (Table 2).

**Table 2:** Sociodemographic characteristics of child caregivers and their intention for their child to receive the COVID-19 vaccination * Fisher’s exact test; ** Chi-square tests for categorical variables; *** t-tests for continuous variables. Significance: p < 0.05

Characteristic		Routine childhood vaccination			
	Total, N (%)	Some, n (%)	All, n (%)	Test	p-value
	230	16 (6.9)	214 (93.1)		
Maternal age, years*					
35-49	25 (10.9)	2 (12.5)	23 (10.8)	0.807	0.807
25-34	88 (38.2)	5 (31.3)	83 (38.8)		
15-24	117 (50.9)	9 (56.3)	108 (50.5)		
Sex of child**					
Male	101 (43.9)	8 (50.0)	93 (43.5)	0.827	0.827
Female	127 (55.2)	8 (50.0)	119 (55.6)		
Missing	2 (0.9)	0 (0)	2 (0.9)		
Parent employed**					
Yes	60 (26.1)	3 (18.8)	57 (26.6)	0.488	0.488
No	170 (73.9)	13 (81.2)	157 (73.4)		
Marital status**					
Single	35 (15.2)	4 (25.0)	31 (14.5)	0.528	0.528
Married	147 (63.9)	9 (56.2)	138 (64.5)		
Previously	48 (20.9)	3 (18.8)	45 (21.0)		
Education*					
None	16 (7.0)	0 (0)	16 (7.5)	0.346	0.346
Primary	108 (47.0)	6 (37.5)	102 (47.7)		
Secondary	106 (46.0)	10 (62.5)	96 (44.9)		
Household wealth quintile**					
Poorest	71 (30.9)	5 (31.3)	66 (30.8)	0.348	0.348
Poor	41 (17.8)	1 (6.3)	40 (18.7)		
Medium	62 (27.0)	3 (18.8)	59 (27.6)		
Wealthy	34 (14.7)	4 (25)	30 (14.0)		
Wealthiest	22 (9.6)	3 (18.8)	19 (8.9)		
Caregiver's intent to receive COVID-19 vaccine*					
No	19 (8.3)	3 (18.8)	16 (7.5)	0.134	0.134
Yes	211 (91.7)	13 (81.3)	198 (92.5)		
Caregiver's age, years***					
Mean ± SD	25.6 (6.1)	24.6 (6.7)	25.7 (6.1)	-0.740	0.460
Child's age, months***					
Mean ± SD	19.2 (13.8)	15.8 (11.8)	19.4 (13.9)	-1.012	0.313
Gravidity***					
Mean ± SD	2.4 (1.6)	2.19 (2.2)	2.42 (1.6)	-0.556	0.579

Table 3 shows that 73.9% (n = 170) of respondents found COVID-19 vaccine benefits very important, while 79.9% (n = 183) had positive family norms for childhood COVID-19 vaccination. For childhood routine vaccinations, 94.7% (n = 218) considered vaccine benefits very important, while 95.2% (n = 217) had positive family norms for childhood COVID-19 vaccination.

**Table 3:** Summary of behavioral and social drivers for COVID-19 and routine childhood vaccination Summary statistics showing frequencies and percentages

COVID-19 vaccination survey			Childhood routine vaccination survey		
Confidence in vaccine benefits	n	%	Confidence in vaccine benefits	n	%
Not at all important	24	10.4	Not at all important	2	0.9
A little important	16	7.0	A little important	2	0.9
Moderately important	20	8.7	Moderately important	8	3.5
Very important	170	73.9	Very important	218	94.7
Family norms			Family norms		
No	46	20.1	No	11	4.8
Yes	183	79.9	Yes	217	95.2
Know where to get vaccination (practical issues)			Know where to get vaccination (practical issues)		
No	51	22.2	No	10	4.4
Yes	179	77.8	Yes	220	95.6
Ease to pay for COVID-19 vaccine (affordability)			Ease to pay for COVID-19 vaccine (affordability)		
Not at all easy	89	38.7	Not at all easy	60	26.1
A little easy	41	17.8	A little easy	50	21.7
Moderately easy	22	9.6	Moderately easy	17	7.4
Very easy	78	33.9	Very easy	103	44.8
Intention for COVID-19 vaccine			Willingness for routine child vaccination		
No, you do not want to	30	13.0	None	0	0
Yes, you do want to	193	83.9	Some	16	6.9
Not sure	7	3.1	All	214	93.1

Table 4 shows BeSD factors associated with childhood COVID-19 vaccination willingness: After controlling for confounders, willingness was three times higher for those with moderately important confidence in vaccine benefits compared to not important at all (PRR: 3.07; 95%CI: 1.37-6.85; p = 0.006), three times higher for very important confidence (PRR: 3.11; 95% CI: 1.41-6.89; p = 0.005), 1.4 times higher with positive family norms (PRR: 1.35; 95% CI: 1.04-1.75; p = 0.022), and 2.7 times higher if the caregiver intended to get vaccinated (PRR: 2.68; 95% CI: 1.18-6.07; p = 0.019).

**Table 4:** Unadjusted and adjusted modified Poisson regression analysis PRR: prevalence rate ratio Significance: p < 0.05

	Unadjusted analysis		Adjusted analysis	
Characteristic	PRR (95%CI)	p-value	PRR (95%CI)	p-value
Confidence in vaccine benefits				
Not at all important	Ref		Ref	
A little important	2.40 (0.95-6.04)	0.063	2.01 (0.83-4.83)	0.121
Moderately important	4.08 (1.83-9.11)	0.001	3.07 (1.37-6.85)	0.006
Very important	4.60 (2.11-10.06)	<0.001	3.11 (1.41-6.89)	0.005
Family norms				
No	Ref		Ref	
Yes	2.05 (1.49-2.81)	<0.001	1.35 (1.04-1.75)	0.022
Know where to get vaccination (practical issues)				
No	Ref		Ref	
Yes	1.13 (0.96-1.33)	0.158	0.9 (0.8-1.02)	0.099
Ease to pay COVID-19 vaccination costs	1.07 (1.02-1.11)	0.003	1.01 (0.98-1.04)	0.634
Maternal age, years	0.95 (0.89-1.03)	0.219	1.04 (0.97-1.11)	0.274
Child’s age	1.01 (0.99-1.01)	0.411	1.01 (0.99-1.01)	0.803
Sex of child				
Male	Ref		Ref	
Female	0.97 (0.87-1.09)	0.613	0.97 (0.89-1.06)	0.512
Gravidity	1.02 (0.99-1.05)	0.128	1.02 (0.99-1.05)	0.193
Household wealth quintile				
Poorest	Ref		Ref	
Poor	0.92 (0.78-1.08)	0.304	1.06 (0.87-1.30)	0.568
Medium	0.89 (0.77-1.03)	0.131	1.03 (0.86-1.22)	0.763
Wealthy	0.91 (0.77-1.09)	0.309	1.02 (0.84-1.23)	0.871
Wealthiest	0.86 (0.67-1.09)	0.208	1.12 (0.94-1.33)	0.206
Caregiver's intent to receive COVID-19 vaccine				
No	Ref		Ref	
Yes	4.25 (1.78-10.19)	0.001	2.68 (1.18-6.07)	0.019
Employment				
Yes	Ref			
No	1.01 (0.89-1.15)	0.888		
Marital status				
Single	Ref			
Married	1.02 (0.86-1.20)	0.833		
Previously married	1.01 (0.83-1.22)	0.955		

## Discussion

This study used the behavioral and social drivers’ framework to assess caregivers’ willingness to vaccinate their children against COVID-19. The results of this study have important implications for vaccination campaigns and highlight three key factors significantly associated with childhood vaccine willingness: caregivers’ confidence in vaccine benefits, caregivers’ own vaccine intentions, and family norms. These insights offer directions for key stakeholders to promote vaccine uptake through targeted messaging and campaigns.

First, caregivers’ confidence in vaccine benefits is strongly associated with willingness to vaccinate children. The health belief model asserts that self-efficacy influences intent, which precedes actual receipt of the vaccine. Therefore, efforts to positively influence confidence in COVID-19 benefits may improve self-efficacy to get their children vaccinated against COVID-19. A previous study conducted in one district in Northern Uganda reported that caregivers’ confidence in the safety of COVID-19 vaccination was a key driver in their willingness to vaccinate [17]. On the contrary, lack of trust in the COVID-19 vaccine was linked to hesitancy [17]. Similar findings in Entebbe, Uganda, revealed fear of vaccine side effects and misinformation as significant barriers, reinforcing the critical need for accurate public health messaging. Parental apathy may be attributed to several factors, such as less effective promotional messaging, exposure to widespread COVID-19 disinformation from low-credibility sources, including mainstream and social media platforms [18]. These factors subsequently lead to societal distrust in COVID-19 vaccines. This finding underscores the critical role of addressing vaccine-related beliefs to enhance uptake. Confidence in vaccine efficacy likely reflects broader trust in the healthcare system and understanding of disease prevention.

Second, caregivers’ own intention to receive the COVID-19 vaccine was associated with a higher likelihood of vaccinating their children. The link between adult vaccination behaviors and their children’s vaccination uptake is consistent with previous studies, highlighting the importance of promoting adult vaccination as a strategy to promote childhood vaccine uptake. For example, in an immunization outreach campaign in rural Southwestern Uganda, caregivers emphasized that vaccinating their children was vital to prevent diseases, reflecting strong beliefs in the protective benefits of vaccines [19]. Another prior study conducted in rural Uganda showed that caregivers' knowledge and attitudes toward immunization were associated with updated and complete childhood vaccination status [20]. This suggests that caregivers who understand the importance or benefits of immunization may be more likely to ensure timely vaccinations for their children. It is worth noting that the COVID-19 pandemic disrupted routine immunization in Uganda, generally hindering access to all existing routine vaccination services. However, early emerging reports revealed that many caregivers prioritized their children's health by seeking immunization services during lockdowns [21]. This association between caregivers' vaccination intentions and child vaccination rates in Uganda highlights the need for comprehensive strategies that empower and inform caregivers, thereby improving child health outcomes.

Third, family norms strongly shaped caregivers’ willingness to vaccinate their children against COVID-19. In Uganda, health decisions are often made collectively within families. When vaccination is accepted within the family, caregivers are more likely to adopt similar attitudes. Social influence, shared information from community leaders or health workers, and trust in family and community networks also play key roles in increasing vaccine acceptance, as vaccination becomes culturally acceptable and credible within the family [22]. Family and community norms have been shown to significantly impact health behaviors such as receiving childhood COVID-19 vaccination [10]. One study found individuals were more willing to receive the COVID-19 vaccine if they believed their community was at risk, highlighting the role of communal norms in health decisions [17]. Another study in Wakiso District, Uganda, identified widespread concerns about vaccine side effects and effectiveness, highlighting the need to address shared norms to improve acceptance [21]. Caregivers’ decisions are often shaped by social influences, including opinions and behaviors of family members. Social approval and community support can enhance the credibility and reinforce the need to get vaccinated, particularly in collectivist settings like Uganda.

Study limitations

First, the cross-sectional design limits the determination of temporal relationships between variables. However, the study provides valuable insights into key factors that are associated with caregivers’ vaccination decisions, grounded in data from a period of heightened interest in COVID-19 vaccine hesitancies. Second, the study was conducted in an urban health facility, which may limit generalizability to other settings. Furthermore, hesitancy toward a novel, pandemic-era vaccine like COVID-19 is plausibly distinct from established childhood routine immunization hesitancy, and while widespread vaccine availability through public-private partnerships may mitigate some access barriers, caregiver attitudes are likely to have evolved since our study implementation. Third, we acknowledge that the study lacked sufficient power to detect smaller effect sizes for other covariates. Fourth, since our study participants were recruited from an urban immunization clinic, the sample likely over-represents caregivers who are already engaged with health services and hold more pro-vaccine attitudes. This selection bias may limit the generalizability of our findings to caregivers who are less engaged with the healthcare system or reside in rural settings. Lastly, social desirability bias may have influenced caregivers’ responses, especially given the prevailing perceptions in the early COVID-19 pandemic period.

Study implications

For practitioners, interventions should focus on addressing safety concerns and emphasizing the benefits of vaccination, using culturally relevant messaging and trusted community influencers. Family-based strategies, such as integrating family-oriented education into healthcare settings, are essential to promote positive vaccination norms. Encouraging caregiver vaccination can increase childhood vaccination rates, and healthcare providers should address both adult and childhood vaccination during visits. Engaging community leaders and sharing real-time data will build trust and reduce fear of side effects. Future research should explore the causal link between caregiver behaviors and vaccination uptake, with urban-rural comparisons to identify context-specific barriers. Behavioral interventions targeting vaccine confidence and social norms should be tested for effectiveness and scalability. Policymakers should support community-driven vaccination initiatives, address cultural norms and misinformation, and invest in education, community engagement, and transparent communication to improve vaccine uptake and ensure equitable access across populations.

## Conclusions

Caregivers’ willingness to vaccinate their children against COVID-19 was associated with their confidence in vaccine benefits, family norms, and caregivers’ own vaccination intentions. Addressing these factors through targeted interventions may help overcome barriers to vaccine uptake, particularly by promoting vaccine confidence and leveraging social influences within families. Focusing on these predictors in future COVID-19 or other vaccination campaigns may improve childhood vaccination rates among urban, healthcare seeking populations in similar settings. By strengthening caregiver trust in vaccines and healthcare systems, these efforts could foster long-term improvements in childhood immunization programs.

## Appendices

Supplementary Table 1. Routine and COVID-19 Immunization Trends, Completion Rates and Parents’ Infant Immunization

Behavior in Kawempe Division, Kampala: Evaluation of the Child Foundation Uganda Immunization

Registry, 2015-2022

Questionnaire in English

Part 1: Demographics

We have a few questions about you and your family.

1. How old are you? _______ (years)

2. What is your attending child’s age? _______ (month/years)

3. Gender of your attending child

   i. Male

   ii. Female

4. How would you describe your work? Circle all that apply.

   i. Job outside of the home

   ii. No job but looking for work

   iii. Student

   iv. Homemaker

   v. Disabled or too ill to work

5. What is your current marital status?

   i. Currently married

   ii. Single (never married)

   iii. Previously married (widowed/divorced/separated)

6. How many times have you ever been pregnant? ________ times

7. How many children do you have? ________

8. What is the highest level of school you completed?

   i. None

   ii. Primary

   iii. Secondary

   iv. University

   v. Others specify _______________

9. Do you own any of these items in your household? Circle all that apply.

i. Radio ii. Television iii. Bicycle iv. Motorcycle v. House vi. Cell phone vii. Computer viii. Indoor

bathroom ix. Water source x. Electricity xi. Car xii. Solar power source

Childhood vaccination survey

Confidence in vaccine benefits (thinking and feeling)

10. How important do you think routine childhood vaccines are for your child’s health? Would you say…

   i. Not at all important

   ii. A little important

   iii. Moderately important

   iv. Very important?

Family norms (social processes)

11. Do you think most of your close family and friends want you to get your child vaccinated with routine

childhood vaccines?

   i. No

   ii. Yes

Intention to get vaccine (motivation)

12. Uganda has a schedule of recommended routine vaccines for children. Do you want your child to get

none of these vaccines, some of these vaccines, or all of these vaccines?

   i. None

   ii. Some

   iii. All

Know where to get vaccination (practical issues)

13. Do you know where to go to get your child routine vaccinated?

   i. No

   ii. Yes

Affordability (practical issues)

14. How easy is it to pay for routine vaccination? When you think about the cost, please consider any

payments to the clinic, the cost of getting there, plus the cost of taking time away from work. Would you

say…

   i. Not at all easy

   ii. A little easy

   iii. Moderately easy, or

   iv. Very easy?

COVID-19 vaccination survey about their child getting the COVID-19 vaccination

Confidence in vaccine benefits (thinking and feeling)

15. How important do you think getting a COVID-19 vaccine will be for your child’s health? Would you

say…

   i. Not at all important

   ii. A little important

   iii. Moderately important

   iv. Very important?

Family norms (social processes)

16. Do you think most of your close family and friends want your child to get a COVID-19 vaccine?

   i. No

   ii. Yes

Intention to get vaccine (motivation)

17. Do you want your child to get a COVID-19 vaccine? Would you say…

   i. No, you do not want to,

   ii. Yes, you do want to

   iii. Not sure?

Know where to get vaccination (practical issues)

18. Do you know where to go to get a COVID-19 vaccine for your child?

   i. No

   ii. Yes

Affordability (practical issues)

19. How easy is it to pay for a COVID-19 vaccination for your child? When you think about the cost,

please consider any payments to the clinic, the cost of getting there, plus the cost of taking time away

from work. Would you say…

   i. Not at all easy

   ii. A little easy

   iii. Moderately easy

   iv. Very easy?

COVID-19 vaccination survey about caregivers getting the COVID-19 vaccination

Confidence in vaccine benefits (thinking and feeling)

20. How important do you think getting a COVID-19 vaccine will be for your health? Would you say…

   i. Not at all important

   ii. A little important

   iii. Moderately important

   iv. Very important?

Family norms (social processes)

21. Do you think most of your close family and friends want you to get a COVID-19 vaccine?

   i. No

   ii. Yes

Intention to get vaccine (motivation)

22. Do you want to get a COVID-19 vaccine? Would you say…

   i. No, you do not want to,

   ii. Yes, you do want to

   iii. Not sure?

Know where to get vaccination (practical issues)

23. Do you know where to go to get a COVID-19 vaccine for yourself?

   i. No

   ii. Yes

Affordability (practical issues)

24. How easy is it to pay for vaccination? When you think about the cost, please consider any payments

to the clinic, the cost of getting there, plus the cost of taking time away from work. Would you say…

   i. Not at all easy

   ii. A little easy

   iii. Moderately easy

   iv. Very easy?
